# Stable Isotopes in Fish Eye Lenses as Potential Recorders of Trophic and Geographic History

**DOI:** 10.1371/journal.pone.0108935

**Published:** 2014-10-03

**Authors:** Amy A. Wallace, David J. Hollander, Ernst B. Peebles

**Affiliations:** College of Marine Science, University of South Florida, St. Petersburg, Florida, United States of America; Phillip Island Nature Parks, Australia

## Abstract

We evaluated eye lenses as potential recorders of stable isotope histories in fish because they consist of metabolically inert optical proteins that are deposited in successive, concentric circles (laminae) much like otolith circuli and tree rings. We conducted four different tests on lenses from red snapper, red grouper, gag, and white grunt. The first test was a low-resolution screening of multiple individuals (4–5 radial groups of laminae per lens, all species except white grunt). Along the radial axis, all individuals exhibited substantial isotopic variability. Red snapper individuals separated into two groups based on *δ* ^15^N and gag separated into two groups based on *δ* ^13^C. Two gag with the greatest variation were chosen for high-resolution temporal analysis using individual laminae from their second eye lenses. The first-order patterns from the high-resolution analysis generally mimicked patterns from the low-resolution screening of grouped laminae, yet the high-resolution plots revealed early-life details that were not apparent in the low-resolution screenings. For the third test, left- versus right-eye variation was compared using high-resolution methods. White grunt left- and right-eye radial isotopic patterns were almost identical for both *δ* ^13^C and *δ* ^15^N, suggesting the variations observed among individual fish were not artifacts. The final test evaluated intra-laminar variation; multiple samples were analyzed from different parts of the same lamina. Seven laminae from three individuals of two species were analyzed in this manner; variations among laminae were found to be much higher than variations within laminae. However, nominal intra-laminar variations were comparable to nominal differences between left and right lenses, suggesting intra-laminar variation established measurement precision. Eye lens isotopes appear to be useful for reconstructing the isotopic histories of individual fish; these histories can be compared with spatially-derived isoscapes to reconstruct individual histories for site fidelity, movement and trophic position.

## Introduction

Internally recorded stable-isotope histories can be used to recreate lifelong trends in animal diets, trophic dependences, and movement within isotopically variable landscapes [1]–[5]. Such lifelong records are difficult to obtain because most tissues undergo metabolic turnover, and this turnover places limits on the retrospective time period that can be investigated [2], [3], [5], [6], [7].

Otoliths, fish scales, and vertebrae are tissues currently used to provide life-history records for fish. Otoliths are used to determine fish age and to reconstruct aquatic environments [8], [9]. Otoliths grow during the entire life of the fish and consist of calcium carbonate deposited within a proteinaceous matrix. Otolith protein is generally not abundant enough (3–4% by weight) to allow retrospective *δ* ^15^N comparisons [10]. Fish scales grow during most of the life of the fish and provide a nonlethal way to measure isotopic ratios. Fish scales overlap old organic material with new material during growth in a phenomenon called “overplating” [11], [12], [13]. Overplating affects isotopic ratios by combining isotopic values from new and old material; scales may not provide a true representation of isotopic information on a temporal scale [11]. Some researchers have explored the use of vertebrae in sharks for stable-isotope analyses with encouraging results [14]–[16]. The collagen extracted from cartilaginous vertebrae is preserved because cartilage has low metabolic activity (little or no turnover) and it deposits in sequential layers [17]. However, this approach cannot be applied to the vertebrae of bony fishes because bone collagen is reworked over time [2], [3]. Although this paper focuses on fish, it should be noted that there are a number of isotopic records for terrestrial animals as well. These include claws, hooves, hair, feathers, and teeth [18], [19]. Although these tissues are isotopically conservative, the period of time represented by the record varies with tissue wear (e.g. claws, hooves) and replacement (e.g. feathers, hair) [18], [19]. Some tissues can be sampled non-invasively on a regular basis to create a long-term isotopic record, but require recapture of the same animal on a regular basis [18], [19].

In this paper, we evaluate fish eye lenses as potential recorders of the isotopic histories of individual fish; the eye lenses of squid (invertebrates) also appear to have this potential [20], [21]. Vertebrate eye lenses are largely composed of different forms of a structural protein, crystallin, that develop in layers (laminae) at the outer lens as the animal grows [22], [23]. During the mid-to-late gastrula stage, lens cells derive from surface ectoderm and differentiate into two cell types, lens epithelial cells and fiber cells [24], [25], although Bloemendal considered all lens cells to be epithelial [26]. At the surface of the lens, epithelial cells form a one-cell-thick outer layer (lens epithelium) adjacent to the fiber cells. Fiber cells remove their cytoplasmic organelles, including the cellular nucleus and its DNA, to establish and maintain optical transparency within the lens [22]–[25], [27]. This process is a specialized form of apoptosis known as “attenuated” apoptosis, where the organelles are removed from the cell over a period of days, leaving behind the crystallin and other proteins [25], [28]. Attenuated apoptosis is different from “classical” apoptosis in which everything, including the cell membrane, is degraded [25].

Fish eye lenses are spherical, and lens fiber cells are laid down in concentric layers much like the layers of an onion [22]–[24]. Fiber cells increase in length as the animal grows, but the increase in length is a step function of lens radius rather than a continuous increase, and this step function creates the physical discontinuities that are visible between adjacent laminae [22], [27]. The oldest part of the lens is at the center (lens nucleus); the nuclear fiber cells are among the oldest cells in the body [25]. The outermost margin of the lens is the youngest material in the lens. *De novo* protein synthesis ceases following attenuated apoptosis [22]. Crystallins and other lens proteins are rich in both carbon and nitrogen, and thus are suitable for *δ* ^13^C and *δ* ^15^N analysis; this contrasts with otoliths, which are nitrogen-poor. Eye lenses thus have the potential to allow reconstruction of lifetime *δ* ^13^C and *δ* ^15^N histories and their associated ecological interpretations.

To investigate variation in fish eye-lens stable isotopes, we selected fish species that occur in geographic locations that had already been the subject of isotopic mapping (isotope maps are known as “isoscapes”). This allowed comparison of any observed isotopic trends within the fish eye lenses with a known range and pattern of isotopic variation documented by Radabaugh et al. [29]. Four species (red snapper, red grouper, gag, white grunt) were selected that have life histories that are geographically bracketed between the polyhaline coastal zone and the outer continental shelf, a region that coincides with the Radabaugh et al. isoscapes (we avoided estuarine, strongly estuary-dependent, and highly migratory species). We were otherwise limited to specimens that became available near the time of analysis, as no specimens were collected for the sole purpose of conducting the present study. We expected trophic positions to increase with age (as mouth gape increases), which generally causes *δ* ^13^C and *δ* ^15^N to increase with age [30], [31].

We conducted four tests to investigate variation in fish eye-lens isotopes (*δ* ^13^C, *δ* ^15^N). First, we conducted a low-resolution comparison of a small number (4–5) of eye-lens layers (groups of laminae) to determine if there was enough isotopic variation during life to warrant further study, with the specific objective being to compare lifetime isotopic variation with isoscapes recently produced by Radabaugh et al. [29], therein possibly providing insight into changes in fish movement and trophic position during life. After discovering that substantial, measurable isotopic variation did exist within the lenses, we used the remaining, second lens to conduct high-resolution comparisons, dissecting (delaminating) the second lens into as many thin layers (hereafter referred to as “laminae”) as was practical, and comparing the high-resolution results with the coarse patterns from the first test. We then compared high-resolution patterns in the left and right lenses of an additional specimen to investigate repeatability. Finally, we examined intra-laminar variation to identify the level of inherent variability (precision) in the method.

## Materials and Methods

### Ethics Statement

No fish were collected or killed for the purpose of this study. All tissues were collected post-mortem according to a protocol (IS00000504) approved by the University of South Florida Institutional Animal Care and Use Committee. None of the sampled species are protected except by recreational and commercial harvest regulations. Fish were obtained from surveys conducted by the Florida Fish and Wildlife Commission (FWC) and from a licensed charter-fishing vessel operating within state and federal regulatory guidelines in waters no more than 80 km distant from John's Pass, Madeira Beach, Florida.

### Lens Collection and Dissection

Four red grouper (*Epinephelus morio*) and three gag (*Mycteroperca microlepsis*) were obtained from an FWC survey conducted 9–10 July, 2013. Eight red snapper (*Lutjanus campechanus*) were obtained from John's Pass charter vessels on 10 September, 2013 and one white grunt (*Haemulon plumierii*) was obtained from a charter vessel on 11 June, 2013. Whole eyes were removed by severing the sclera at its junction with the optic nerve and by severing the rectus (orbital) muscles near their junction with the sclera. Eyes were wrapped in aluminum foil, placed in plastic bags on ice, and frozen at −40°C upon return to the laboratory.

Eyes were thawed individually before dissection. After thawing, an incision was made with a scalpel to create a flap in the cornea, which was folded back to allow removal of the lens using forceps. Exterior tissue and vitreous material were manually removed using a deionized water rinse. The rinsed lens, which contained the lens nucleus, cortex, and lens epithelium together as one cohesive unit (whole lens), was then placed on a glass petri dish where successive layers of cortical laminae were separated using two pairs of fine-tip forceps under a dissecting stereomicroscope, leaving the lens nucleus as the final tissue in the analyzed series. The lens nucleus did not visibly delaminate further when crushed. Because the total number of cortical laminae varied with lens diameter (age), the convention for numbering laminae started with the lens nucleus as 1, with assigned numbers increasing toward the outer lens margin. Lamina removal (delamination) started near one lens pole and proceeded to the other, always starting the removal process at the same pole (note that the “anterior-posterior” polar axis referred to in studies of the human eye applies to forward-oriented eyes; most fish have laterally oriented eyes, and thus the homolog of the human anterior-posterior polar axis is usually mediolateral in fish, with the anterior pole and associated primary sutures being oriented laterally). Some forms of crystallin are water-soluble [26], [32]; deionized water was used sparingly to assist delamination.

After each delamination, an ocular micrometer was used to measure the diameter at the equator (to nearest 0.1 mm); the equator was identified as the largest diametric measurement that could be made perpendicular to the (mediolateral) polar axis. Lamina position is the radial midpoint of the lamina (in mm) on the equator, where the midpoint is the lens radius after lamina removal plus half the thickness of the removed lamina.

### Lens Sample Processing

Four types of isotopic variation were plotted: 1) low-resolution screening plots, 2) high-resolution plots for selected, screened individuals, 3) plots of left vs. right eyes, and 4) plots of variation within individual laminae (intra-laminar variation). For low-resolution screening, eye lenses were separated into 4–5 sections using fine-tip forceps, with multiple laminae intentionally included within each section; the number of true laminae constituting each section was not known. For all other tests, an effort was made to subdivide the lenses into the smallest thicknesses that were manually practical (“laminae”). Individual sections and laminae were stored separately in 1-dram glass vials. Laminae became desiccated in <1 h at 25°C, after which most samples were homogenized using a mortar and pestle; exceptions were laminae that were haphazardly selected for intra-laminar variation analysis, which were subdivided first and then homogenized.

Dried laminae were weighed to the nearest µg on an analytical balance. A dry weight of 300–600 µg of material was placed in tin capsules for combustion and isotopic analysis. ^13^C/^12^C and ^15^N/^14^N and C:N were measured in replicate using a Carlo-Ebra NA2500 Series II elemental analyzer coupled to a continuous-flow ThermoFinnigan Delta+XL isotope ratio mass spectrometer at the University of South Florida College of Marine Science in St. Petersburg, Florida. The lower limit of quantification was 12 µg C or N. Calibration standards were NIST 8573 and NIST 8574 L-glutamic acid standard reference materials. Analytical precision, obtained by replicate measurements of NIST 1577b bovine liver, was ±0.11 ‰ for *δ* ^13^C and ±0.19 ‰ for *δ* ^15^N (average standard deviations of n = 30 replicates). Results are presented in standard notation (*δ*, in ‰) relative to international standards Pee Dee Belemnite (PDB) and air:
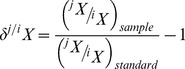
 where *X* is the element and *j* and *i* are each an isotope of *X*.

### Data Location and Analysis

All data are published at the Gulf of Mexico Research Initiative Information and Data Cooperative (https://data.gulfresearchinitiative.org/data/R1.x135.120:0006). In all comparisons except for the intra-laminar comparison, replicate isotope measurements were averaged and plotted for descriptive purposes. In the intra-laminar comparison, the objective was to determine if inter-laminar variation was greater than intra-laminar variation; individual replicate isotope measurements were compared among laminae using one-way ANOVA. Individual laminae were compared using 95% Tukey HSD intervals as a multiple range test (Statgraphic Centurion, v. 16.2.04). In all comparisons, means and replicates were plotted without any exclusion.

## Results

**Figure 1:**
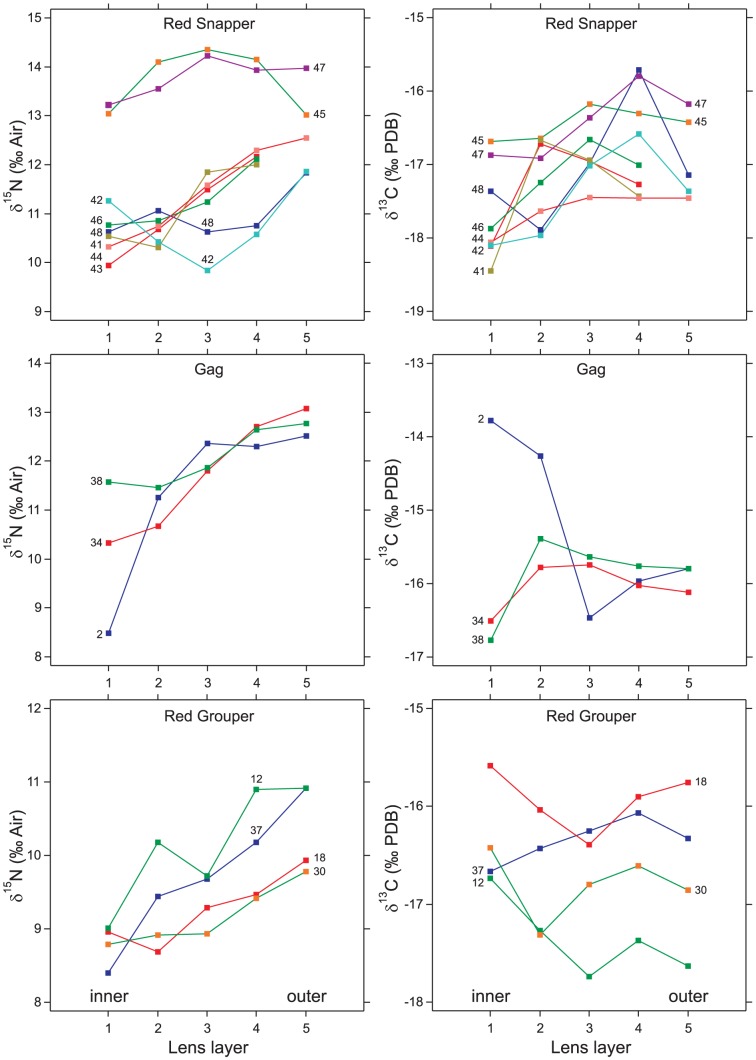
Low-resolution screening. Rapid screening of specimens to detect large-scale differences in isotopic history. Each plotted line is a different specimen; lines are labeled with specimen numbers.

Low-resolution screening analyses were conducted for eight red snapper, four red grouper, and three gag. All individuals had variable isotopic values among lens layers (Figure 1). The majority of values were centered within ranges reported by Radabaugh et al. [29] for trawl-caught demersal fishes on the West Florida Shelf (−19– −14 ‰ *δ* ^13^C; 7–13.5‰ *δ* ^15^N). Red snapper appeared to form two groups based on their δ^15^N values; fish 45 and 47 (Figure 1) had consistently high *δ* ^15^N values. There were no clear groupings in red grouper. Gag 2 had higher *δ* ^13^C values for its innermost layer than the other two gag, and all three gag had isotopic values that converged in the outermost layers.

Gag 2 and 38 were analyzed using high-resolution analysis to further evaluate the difference recorded in the innermost layers. The second lens from gag 2 (3.1 mm radius) was dissected into 14 laminae plus a lens nucleus of 0.65 mm radius, indicating an average laminar thickness of 175 µm. The second lens from gag 38 (3.2 mm radius) was dissected into 16 laminae plus a lens nucleus of 0.60 mm radius, indicating an average laminar thickness of 163 µm. When data from the two lenses were combined, dry weight (*DW*, in mg) increased as *DW* = 3.24*r* ^3^ (*n* = 32, *R* ^2^ = 0.99, *p*<0.0001), where *r* is lens radius in mm. The mean dry density of these two gag lenses was 0.89 mg mm^−3^. Laminar thickness varied at a sub-annual scale in these two specimens, but did not have a strong positive or negative overall relationship with lens radius (slope *p*>0.05).

**Figure 2:**
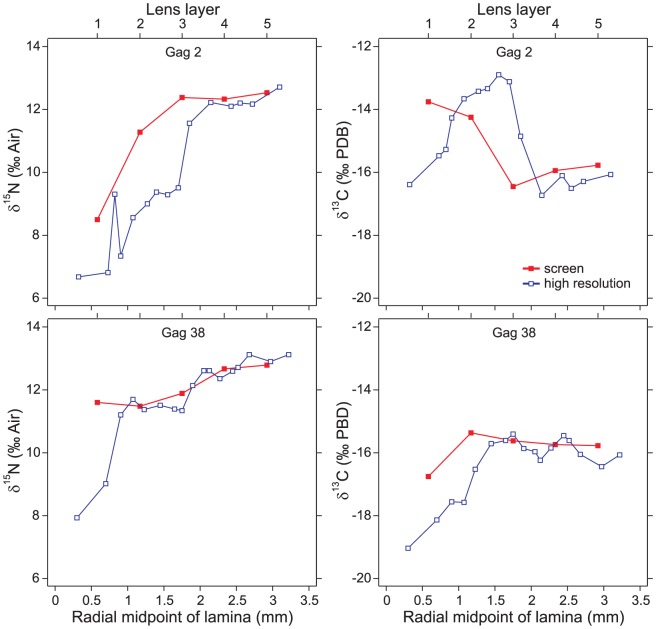
Low- and high-resolution comparison. The low-resolution screening method (analysis of coarse lens layers) is compared with the high-resolution method (analysis of individual laminae) for gag 2 (upper panels) and gag 38 (lower panels). The leftmost observation in the high-resolution method is for the lens nucleus; the remaining observations are for individual laminae. Gag 2 was 704 mm total length (TL); gag 38 was 832 mm TL. Both were female.

The exponential (cubic) increase in average laminar weight that accompanies increasing lens size translates into a mass-balanced bias toward outer laminae when groups of laminae are processed together as a collective layer (e.g., during low-resolution screening analysis). The innermost layer from the screening analysis was most likely to be misrepresentative of patterns observed in the high-resolution analysis (Figure 2). Nevertheless, the screening method indicated different incipient *δ* ^15^N values for gag 2 and 38, and this difference was borne out by the high-resolution analysis, albeit at a nominally lower difference (2.5 vs. 3.0 ‰).

**Figure 3:**
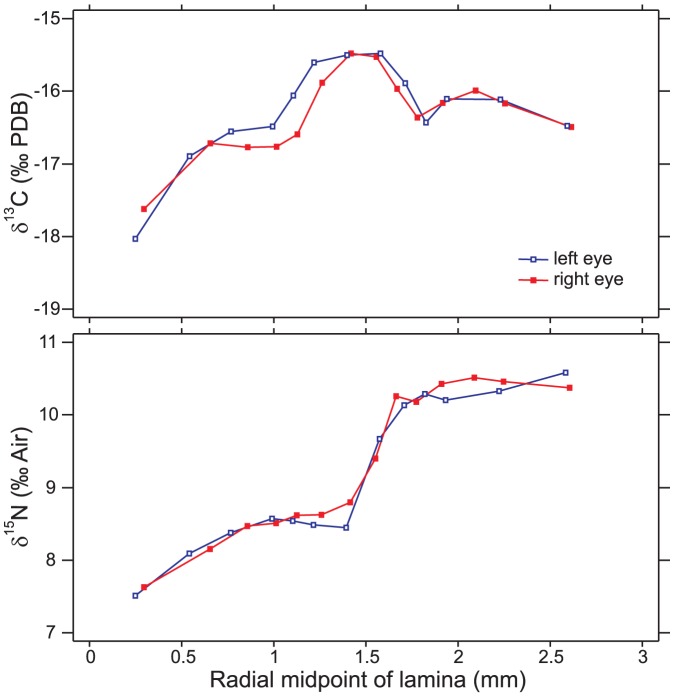
High-resolution comparisons of left and right eyes of white grunt specimen 2.

**Figure 4:**
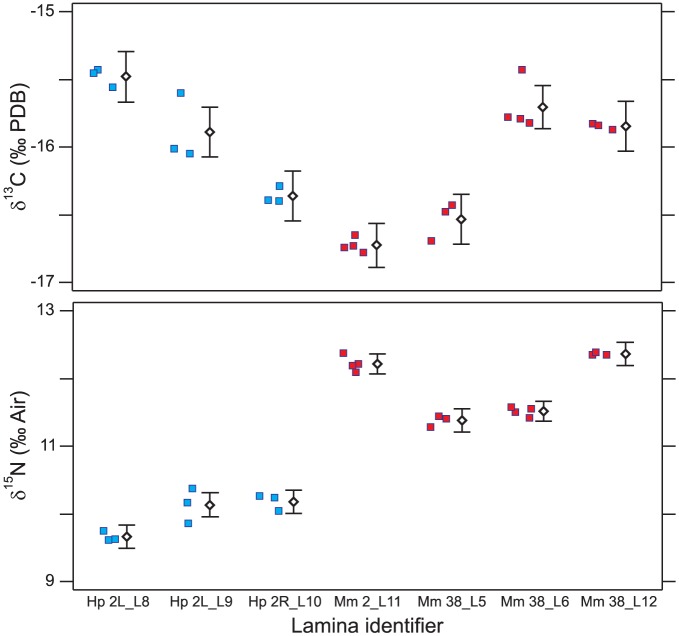
Intra- and inter-laminar comparison. Comparison of intra- and inter-laminar isotopic variation in white grunt specimen 2, left eye (Hp 2L) and right eye (Hp 2R) and gag specimens 2 and 38 (Mm 2 and Mm 38). Lamina number (e.g., L8  =  lamina 8) is indicated within lamina identifiers. Data points have been horizontally jittered for clarity, and are summarized by means (diamonds) with 95% Tukey HSD intervals.

The left lens of white grunt 2 was dissected into 12 laminae plus the lens nucleus. The radius of the left lens nucleus was 0.50 mm and the radius of the whole left lens was 2.85 mm, indicating a mean laminar thickness of 196 µm. The right lens was dissected into 13 laminae plus the lens nucleus. The radius of the right lens nucleus was 0.60 mm and the radius of the whole right lens was 2.88 mm, indicating a mean laminar thickness of 175 µm. Isotopes in both eyes were centered within ranges reported by Radabaugh et al. [29], and *δ* ^15^N increased during life; this latter trend was observed in 15 of the 16 fish examined (94%, with red snapper 45 being the sole exception). Isotopic patterns were very similar between left and right eyes (Figure 3), yet plotted pairings of radial midpoints in Figure 3, average laminar thicknesses (196 vs. 175 µm), and the difference in total lamina count (12 vs. 13) were not in perfect agreement. One-way ANOVA indicated inter-laminar variation (Figure 4) was much larger than intra-laminar variation for both *δ* ^13^C [*F*(6,16)  = 40.6, *p*<0.0001] and *δ* ^15^N [*F*(6,16)  = 232.4, *p*<0.0001].

A total of 197 samples were analyzed (72 sections, 125 laminae). The range of mass C:N was 2.72–3.37. The mass C:N mean value was 2.97±0.12 (molar C:N range = 3.17–3.39; mean = 3.46±0.14). The C:N ratio was consistently <3.5; therefore, no lipid correction or normalization was needed because the lipid concentrations were uniformly low [33].

## Discussion

The results indicate low-resolution isotopic screening has strong potential as an index of broad-scale changes in the isotopic history of individual fish. Broad-scale changes were evident in all species, yet red snapper and gag appeared to originate from distinct groups that converged later in life (all fish were collected from the same geographic region). The magnitude of isotopic variation observed in both the low- and high-resolution tests (Figures 1–3) matches the scale of variation in isoscapes produced by Radabaugh et al. [29] for the West Florida Shelf, the region from which the fish in our tests were collected. The low-resolution screening tests were rapid and cost-effective, but sometimes missed important life events. In the case of the gag (Figure 2), the missed detail involved early life history; for gag 2, the low-resolution analysis had a starting value of −13.8 ‰ for *δ* ^13^C while the high-resolution analysis was variable, starting at −16.4 and increasing to −12.9 ‰. The high-resolution approach contains the most information that can be obtained practically using the manual delamination method.

The white grunt's left and right eyes identified very similar lifelong trends (Figure 3), suggesting the temporal trends observed in the other comparisons (e.g., Figure 1) were repeatable and were not artifacts. The scale of variability between left and right eyes was similar to that of intra-laminar variation. Most intra-laminar variation was less than the instrument error calculated using bovine liver (±0.11 ‰ for δ^13^C; ±0.19 ‰ for δ^15^N,). Small, intra-laminar variation likely imparts a limit to the precision of the overall method.

All of the values and trends in the eye-lens isotopes are consistent with expected life-history patterns and trophic positions, as interpreted using published *δ* ^13^C and *δ* ^15^N isoscapes for demersal fish muscle [29]. Isoscapes visually portray spatial isotopic variation within natural systems [34]. While many terrestrial isoscapes exist, a need exists for developing more marine isoscapes [35]. When eye-lens isotopes are compared to observed and modeled isoscapes for demersal fish muscle on the West Florida Shelf [29], [36], it appears that the two red snapper with higher *δ* ^15^N (Figure 1) traveled from the northern end of a *δ* ^15^N latitudinal gradient, and those with lower *δ* ^15^N traveled from or remained within more southerly waters. Isoscape trends in *δ* ^13^C of demersal fishes are more depth-related (higher values in shallow water) than latitude-related [29]. Red snapper eye-lens *δ* ^13^C remained below −15.5‰ throughout life, which is consistent with the red snapper's known preference for deeper water on the middle and outer portions of West Florida Shelf. The three gag may have started life at different locations, but spent a lengthy period of time together before collection. Unlike the red snapper, the gag uses shallow inshore waters as nursery habitat [37]. The elevated *δ* ^13^C in gag 2 (Fig. 2) is indicative of residence in shallow water. However, because *δ* ^13^C for demersal fish in inshore waters varies along the coastline as a statistical function of water clarity/depth (photosynthetically active radiation at bottom), particulate organic carbon concentration, and sea-surface temperature [36], not all gag can be expected to reside in high-*δ* ^13^C locations while in shallow nursery habitats.

All of the preceding geographic interpretations are subject to future corrections that accommodate shifts in trophic level and associated changes in total trophic fractionation. The increase in *δ* ^15^N that was observed in most (94%) specimens is consistent with increasing trophic level. The trend in the sole exception, red snapper 45, would be explained if the fish moved south later in life [29], [36]. Among the four species, the gag (a large, piscivorous grouper) had relatively high *δ* ^15^N values at capture; this is consistent with expected differences in trophic position among species.

Directions for future work include, but are not limited to, the following areas of study: 1) eye lens aging by synchronizing lamina radial position with otolith-based or known captive age; relationships between fish eye-lens diameter and age, lens wet-weight and age, and lens dry-weight and age have already been established [38]–[41], 2) validating diet relationships using compound specific stable isotope analysis of the both the trophic and source amino acids within different laminae [42]–[44], 3) determination of long-term geographic histories for individual fish by combining isotope histories, knowledge of how trophic level changes with age (trophic growth rate), and output from isoscape models [36]; modeled isoscape outputs must be synchronized with time periods within eye lenses for accuracy because spatial stationarity cannot be assumed [36], [45], 4) comparing isotopic fractionation of eye lens tissues with fractionation of other tissues [46], [47]. Studying captive fish may be particularly definitive both for aging eye lenses and for validating diet relationships with eye-lens isotopes. However, the production and accurate interpretation of long-term geographic histories for individual fish require that eye-lens aging and trophic growth rate studies be completed first.

While our ability to rigorously interpret geographic histories from the eye-lens isotope data is limited by the present lack of the aforementioned studies, the patterns of variation within the eye lenses (Figs. 1–4) indicate that the overall approach is promising. There is no other known source of simultaneous *δ* ^13^C and *δ* ^15^N temporal records within bony fish. The approach presented here is not necessarily limited to bony fish, and may also be applied to other vertebrates as long as the spatiotemporal distribution of apoptosis within the lens is taken into consideration. In fish, TUNEL assays (terminal deoxynucleotidyl transferase dUTP nick-end labeling) indicate that even recently formed laminae can be apoptotic and thus isotopically conservative [24], whereas humans and other higher vertebrates may contain large proportions of pre-apoptotic (potentially non-conservative) cells in the outer cortical region of the lens.

## References

[1] Tieszen LL, Boutton TW, Tesdahl KG, Slade NA (1983) Fractionation and turnover of stable carbon isotopes in animal tissues: implications for *δ* ^13^C analysis of diet. Oecologia 57(1–2): 32–37. 28310153 10.1007/BF00379558

[2] Hobson KA, Clark RG (1992) Assessing avian diets using stable isotopes I: Turnover of ^13^C in tissues. Condor 94(1): 181–188.

[3] Hobson KA, Clark RG (1992) Assessing avian diets using stable isotopes II: Factors influencing diet-tissue fractionation. Condor 94(1): 189–197.

[4] Post DM (2002) Using stable isotopes to estimate trophic position: Models, methods, and assumptions. Ecology 83(3): 703–718.

[5] Phillips DL, Eldridge PM (2006) Estimating the timing of diet shifts using stable isotopes. Oecologia 147(2): 195–203. 16341714 10.1007/s00442-005-0292-0

[6] Thompson RC, Ballou JE (1956) Studies of metabolic turnover with tritium as a tracer: V. The predominantly non-dynamic state of body constituents in the rat. J Biol Chem 223(2): 795–809. 13385227

[7] Fry B, Arnold C (1982) Rapid C-13/C-12 turnover during growth of brown shrimp (*Penaeus aztecus*). Oecologia 54(2): 200–204. 28311429 10.1007/BF00378393

[8] Jones CM (1986) Determining age of larval fish with the otolith increment technique. Fish Bull 84: 91–103.

[9] Thorrold SR, Campana SE, Jones CM, Swart PK (1997) Factors determining *δ* ^13^C and δ^18^O fractionation in aragonitic otoliths of marine fish. Geochim Cosmochim Acta 61(14): 2909–2919.

[10] Campana SE (1999) Chemistry and composition of fish otoliths: pathways, mechanisms and applications. Mar Ecol Prog Ser 188: 263–297.

[11] Hutchinson JJ, Trueman CN (2006) Stable isotope analyses of collagen in fish scales: limitations set by scale architecture. J Fish Biol 69: 1874–1880.

[12] Trueman CN, Moore A (2007) Use of the stable isotope composition of fish scales for monitoring aquatic ecosystems. In: Dawson TE, Siegwolf RTW, (eds.)Terrestrial EcologyVolume1..

[13] Woodcock SH, Walther BD (2014) Trace elements and stable isotopes in Atlantic tarpon scales reveal movements across estuarine gradients. Fish Res 153: 9–17.

[14] Estrada JA, Rice AN, Natanson LJ, Skomal GB (2006) Use of isotopic analysis of vertebrae in reconstructing ontogenetic feeding ecology in white sharks. Ecology 87(4): 829–834. 16676526 10.1890/0012-9658(2006)87[829:uoiaov]2.0.co;2

[15] Kerr LA, Andrews AH, Cailliet GM, Brown TA, Coale KH (2006) Investigations of Δ^14^C, *δ* ^13^C, and *δ* ^15^N in vertebrae of white shark (*Carcharodon carcharias*) from the eastern North Pacific Ocean. Environ Biol Fishes 77(3–4): 337–353.

[16] Werry JM, Lee SY, Otway NM, Hu Y, Sumpton W (2011) A multi-faceted approach for quantifying the estuarine-nearshore transition in the life cycle of the bull shark, *Carcharhinus leucas* . Mar Freshw Res 62(12): 1421–1431.

[17] Walker JL, Macko SA (1999) Dietary studies of marine mammals using stable carbon and nitrogen isotopic ratios of teeth. Mar Mamm Sci 15(2): 314–334.

[18] Dalerum F, Angerbjörn A (2005) Resolving temporal variation in vertebrate diets using naturally occurring stable isotopes. Oecologia 144: 647–658. 16041545 10.1007/s00442-005-0118-0

[19] Ethier DM, Kyle CJ, Kyser TK, Nocera JJ (2010) Variability in the growth patterns of the cornified claw sheath among vertebrates: implications for using biogeochemistry to study animal movement. Can J Zool 88: 1043–1051.

[20] Parry MP (2003) The trophic ecology of two ommastrephid squid species, *Ommastrephes bartramii* and *Sthenoteuthis oualaniensis*, in the North Pacific sub-tropical gyre. Doctoral dissertation, University of Hawaii at Manoa.

[21] Hunsicker ME, Essington TE, Aydin KY, Ishida B (2010) Predatory role of the commander squid *Berryteuthis magister* in the eastern Bering Sea: Insights from stable isotopes and food habits. Mar Ecol Prog Ser 415: 91–108.

[22] Nicol JAC (1989) The eyes of fishes. Oxford: Oxford University Press. 308p.

[23] Horwitz J (2003) Alpha-crystallin. Exp Eye Res 76(2): 145–153. 12565801 10.1016/s0014-4835(02)00278-6

[24] Dahm R, Schonthaler HB, Soehn AS, van Marle J, Vrensen GFJM (2007) Development and adult morphology of the eye lens in the zebrafish. Exp Eye Res 85(1): 74–89. 17467692 10.1016/j.exer.2007.02.015

[25] Wride MA (2011) Lens fibre cell differentiation and organelle loss: many paths lead to clarity. Philos Trans R Soc Lond B Biol Sci 366(1568): 1219–1233. 21402582 10.1098/rstb.2010.0324 PMC3061109

[26] Bloemendal H (1982) Lens proteins. CRC Crit Rev Biochem 12(1): 1–38. 7037295 10.3109/10409238209105849

[27] Vihtelic TS (2008) Teleost lens development and degeneration. Int Rev Cell Mol Biol 269: 341–373. 18779061 10.1016/S1937-6448(08)01006-X

[28] Dahm R (1999) Lens fibre cell differentiation—a link with apoptosis? Ophthalmic Res 31(3): 163–183. 10224500 10.1159/000055530

[29] Radabaugh KR, Hollander DJ, Peebles EB (2013) Seasonal *δ* ^13^C and *δ* ^15^N isoscapes of fish populations along a continental shelf trophic gradient. Cont Shelf Res 68: 112–121.

[30] Karpouzi VS, Stergiou KI (2003) The relationships between mouth size and shape and body length for 18 species of marine fishes and their trophic implications. J Fish Biol 62: 1353–1365.

[31] McMahon KW, Hamady LL, Thorrold SR (2013) Ocean ecogeochemistry: a review. Oceanography and Marine Biology: An Annual Review 51: 327–373.

[32] Sharma KK, Santhoshkumar P (2009) Lens aging: Effects of crystallins. Biochim Biophys Acta 1790(10): 1095–1108. 19463898 10.1016/j.bbagen.2009.05.008 PMC2743770

[33] Post DM, Layman CA, Arrington DA, Takimoto G, Quattrochi J, et al (2007) Getting to the fat of the matter: models, methods and assumptions for dealing with lipids in stable isotope analyses. Oecologia 152: 179–189. 17225157 10.1007/s00442-006-0630-x

[34] West JB, Bowen GJ, Dawson TE (2010) Preface: Context and background for the topic and book. In: West JB, Bowen GJ, Dawson TE, Tu KP, editors. Isoscapes: Understanding movement, pattern, and process on Earth through isotope mapping. New York: Springer. pp. v–xi.

[35] Hobson KA, Barnett-Johnson R, Cerling T (2010) Using isoscapes to track animal migration. In: West JB, Bowen GJ, Dawson TE, Tu KP, editors. Isoscapes: Understanding movement, pattern, and process on Earth through isotope mapping.New York:Springer pp. 273–298.

[36] Radabaugh KR, Peebles EB (2014) Multiple regression models of *δ* ^13^C and *δ* ^15^N for fish populations in the eastern Gulf of Mexico. Cont Shelf Res 84: 158–168.

[37] Fitzhugh GR, Koenig CC, Coleman FC, Grimes CB, Sturges W (2005) Spatial and temporal patterns in fertilization and settlement of young gag (*Mycteroperca microlepis*) along the West Florida Shelf. Bulletin of Marine Science 77(3): 377–396.

[38] Burkett RD, Jackson WB (1971) The eye lens as an age indicator in freshwater drum. Am Midl Nat 85(1): 222–225.

[39] Siezen RJ (1989) Eye lens aging in the spiny dogfish (*Squalus acanthias*): 1. Age-determination from lens weight. Curr Eye Res 8(7): 707–712. 2791619 10.3109/02713688909025805

[40] Al-Hassan LAJ, Al-Sayab AA (1994) Eye lens diameter as an age indicator in the catfish, *Silurus triostegus* . Pak J Zool 26(1): 81–82.

[41] Al-Mamry J, Jawad L, Al-Busaidi J, Al-Mamari A, Al-Mamry S, et al (2012) The use of eye lens diameter and weight in age determination in *Siganus canaliculatus* (Park, 1797) (Perciformes, Siganidae) collected from the Arabian Sea coasts of Oman. Natura Montenegrina 11(1): 73–78..

[42] Popp BN, Graham BS, Olson RJ, Hannides C, Lott MJ, et al. (2007) Insight into the trophic ecology of yellowfin tuna, *Thunnus albacares*, from compound-specific nitrogen isotope analysis of proteinaceous amino acids. In: Dawson TE, Siegwolf RTW, editors. Terrestrial Ecology, Volume I. Elsevier pp. 173–190.

[43] Chikaraishi Y, Ogawa NO, Kashiyama Y, Takano Y, Suga H, et al (2009) Determination of aquatic food-web structure based on compound-specific nitrogen isotopic composition of amino acids. Limnol Oceanogr Methods 7: 740–750.

[44] Ellis G (2012) Compound-specific stable isotopic analysis of protein amino acids: Ecological applications in modern and ancient systems. Doctoral dissertation, University of South Florida, Tampa, FL.

[45] Haché S, Hobson KA, Bayne EM, Van Wilgenburg SL, Villard MA (2014) Tracking natal dispersal in a coastal population of a migratory songbird using feather stable isotope (*δ* ^2^H, *δ* ^34^S) tracers. PLOS ONE DOI:10.1371/journal.pone.0094437. 10.1371/journal.pone.0094437 PMC3989223 24740314

[46] Caut S, Angulo E, Courchamp F (2009) Variation in discrimination factors (Δ^15^N and Δ^13^C): the effect of diet isotopic values and applications for diet reconstruction. J Appl Ecol 46: 443–453.

[47] Martinez del Rio C, Carleton SA (2012) How fast and how failthful: the dynamics of isotopic incorporation into animal tissues. J Mammal 93(2): 353–359.

